# Mutational Landscape of Gastric Adenocarcinoma of the Fundic Gland Type Revealed by Whole Genome Sequencing

**DOI:** 10.1002/cam4.70290

**Published:** 2024-10-09

**Authors:** Hu Wei, Ze Min Chen, Xiu Fen Xue, Li Xia Xi, Gen Hua Yang, Zhi Yong Zhai, Zhao Yu Huang, Ping Zhou, Chong Ju Bao, Li Juan You, Mei Ping Ou Yang, Gui Li Xia, Zhi Yu Zeng, Xiao Bing Cui, Xiao Juan Pei, Wei Gong

**Affiliations:** ^1^ Department of Gastroenterology, Shenzhen Hospital Southern Medical University Shenzhen Guangdong China; ^2^ The Third School of Clinical Medicine Southern Medical University Shenzhen Guangdong China; ^3^ Department of Anaesthesia and Intensive Care and Peter Hung Pain Research Institute The Chinese University of Hong Kong Hong Kong, SAR China; ^4^ Department of Pathology, Shenzhen Hospital Southern Medical University Shenzhen Guangdong China

**Keywords:** gastric adenocarcinoma, gastric adenocarcinoma of the fundic gland type, *GNAS*, mutational landscape, whole genome sequencing

## Abstract

**Background:**

Gastric adenocarcinoma of the fundic gland type (GA‐FG) is a newly described variant of gastric adenocarcinoma with lack of knowledges regarding its genetic features.

**Methods:**

We performed whole‐genome sequencing (WGS) in formalin‐fixed paraffin‐embedded (FFPE) tumor tissues and matched adjacent noncancerous specimens from 21 patients with GA‐FG, and integrated published datasets from 1105 patients with traditional gastric adenocarcinoma with the purpose of dissecting genetic determinants both common to conventional gastric adenocarcinoma and unique to GA‐FG disease.

**Results:**

We characterized the genomic architecture of GA‐FG disease, revealing the predominant proportion of C > T substitution among the six types of SNVs. *GNAS* was the most significantly mutated driver gene (14.29%). 42.8% of samples harbored “Kataegis.” Distinct genomic alterations between GA‐FG and conventional gastric cancer were identified. Specifically, low mutational burden and relatively moderate mutational frequencies of significantly mutated driver genes, coupled with the absence of non‐silent alterations of formerly well‐known drivers such as *TP53*, *PIK3CA* and *KRAS* were identified in GA‐FG patients. Oncogenic signaling pathway analysis revealed mutational processes associated with focal adhesions and proteoglycans in cancer, highlighting both common and specific procedures during the development of GA‐FG and conventional gastric cancer.

**Conclusion:**

Our study is the first to comprehensively depict the genomic landscape highlighting the multidimensional perturbations in GA‐FG patients. These discoveries offered mechanistic insights for novel diagnostic and therapeutic strategies for patients with such disease.

## Graphical abstract

This study characterized the genomic architecture of GA‐FG disease. We identified distinct genomic alterations between GA‐FG and conventional gastric cancer. We uncovered oncogenic signaling pathways both common to traditional gastric adenocarcinomas and unique to GA‐FG patients.

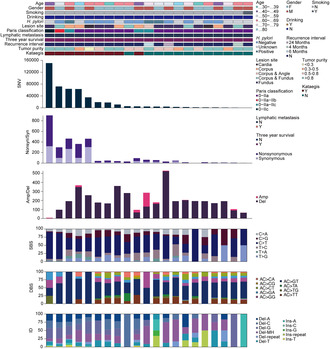

**Abbreviations**
ACRG: Asian Cancer Research Group
CNAs: Copy number alterations
CRC: Colorectal cancers
DBS: doublet‐base substitutions
Del‐MH: Deletions with microhomology
FFPE: formalin‐fixed paraffin‐embedded
GA‐FG: Gastric adenocarcinoma of the fundic gland type
*H. pylori*
: *Helicobacter pylori*
ICGC: International Cancer Genome Consortium
ICI: immune checkpoint inhibitor
ID: insertion‐and‐deletion
IDA: Iron deficiency anemia
Mb: Megabase
PARPi: Poly (adenosine diphosphate ribose) polymerase (PARP) inhibitors
SMGs: Significantly mutated genes
SNVs: Somatic single‐nucleotide variants
TMB: Tumor mutational burden
WGS: Whole‐genome sequencing

## 1 Introduction

Gastric adenocarcinoma of the fundic gland type (GA‐FG) originated from the gastric mucosa of the fundic gland region is a new, rare subtype of gastric neoplasm, and has been formally included as a variant of gastric adenocarcinoma in the latest version of the WHO classification of digestive tumors issued in 2019 [1, 2]. As a well‐differentiated tubular adenocarcinoma with submucosal invasion, GA‐FG is defined as a neoplastic lesion consisted of clustered glands and irregular branching cords of oxyntic epithelium. Most of them are predominated by chief cell, parietal cells, or an even admixture of both cell types [3]. The first case of GA‐FG was described by Tsukamoto et al. [4, 5]. To date, over 300 cases have been reported worldwide [6, 7, 8]. Although most early cases were confined to Japanese and South Korean, non‐Asian patients, including African‐Americans, Caucasians and Hispanics have been recently published [3], suggesting that such neoplasm can arise in multiethnic populations. Also, the proportion of gastric adenocarcinomas diagnosed as GA‐FG is expected to increase, owing to the global endeavors in *Helicobacter pylori* (*H. pylori*) eradication.

GA‐FG has distinct clinicopathological features from conventional gastric cancer, especially in terms of tumor locations, histological characteristics, and phenotypic expression [9]. For example, chronic *H. pylori* infection with the stepwise Correa's cascade is the main risk factor for the carcinogenesis of common gastric adenocarcinoma [10, 11]. However, *H. pylori* and the associated intestinal metaplasia and mucosal atrophy are always absent in most cases of GA‐FG [6]. Likewise, iron deficiency anemia (IDA), a phenomenon highly prevalent in common gastric cancer [12], is rarely observed in GA‐FG patients. Moreover, in contrast to its counterparts, GA‐FG is classified as a type of low‐grade malignancy in view of their mild atypia, characterized by rare lymphatic and venous invasion, low proliferative activity, and a lack of recurrence and metastasis. Patients with GA‐FG treated by endoscopic resection are considered to have a favorable prognosis, although long‐term follow‐up surveillance is still necessary [7]. Considering the definition, clinicopathological features and treatment strategies for GA‐FG have not yet been established, a comprehensive analysis to dissect the molecular signatures of this neoplasm is thus demanded.

Here, we delivered the first, to the best of our knowledge, systematical mutational landscape of GA‐FG by performing whole‐genome sequencing (WGS) in formalin‐fixed paraffin‐embedded (FFPE) tumor tissues and matched adjacent noncancerous specimens from 21 patients. We identified the underlying genetic abnormalities of this disease, including distributions and frequency of all mutations, somatic single‐nucleotide variants (SNVs), tumor mutational burden (TMB), and significantly mutated genes (SMGs) with driver potential. Furthermore, we integrated published datasets from 1105 patients with gastric adenocarcinoma extracted from 3 cohorts, as well as our GA‐FG cohort. We herein unraveled genomic signatures both unique to GA‐FG patients and common to GA‐FG and gastric cancer disease. Collectively, our findings may offer novel therapeutic target potential for the diagnosis, prognosis, and treatment of this neoplasm.

## 2 Materials and Methods

### 2.1 Sample Collection

FFPE samples were obtained from 21 enrolled GA‐FG patients who underwent endoscopic surgery at Shenzhen Hospital, Southern Medical University from December 2015 to April 2021. GA‐FG was diagnosed by experienced endoscopists, then confirmed by two independent pathologists via immunohistochemistry. None of these patients received preoperative chemotherapy or radiotherapy prior to sample collection. Matched adjacent noncancerous samples were defined as tissues with normally histological morphology and ≥ 3 cm away from the tumor tissues. All protocols were approved by the Ethic Committee of Southern Medical University (NYSZYYEC20190013) after obtaining patients' informed consent.

### 2.2 DNA Extraction, Library Construction and Whole‐Genome Sequencing

DNA extracted from FFPE samples using the QIAamp DNA FFPE Tissue Kit (Qiagen, Hilden, Germany) according to the manufacture's instruction. DNA quantity was assessed by Nanodrop 2000 spectrophotometer and Qubit 2.0 Fluorometer with Quanti‐IT dsDNA HS Assay Kit (Thermo Fisher Scientific, MA, USA). The sequencing libraries were further constructed by DNA Library Prep Kit (Vazyme, Nanjing, China). Geneplus‐2000 sequencing platform (Geneplus, Beijing, China) sequenced the libraries in a 150 bp paired‐end manner with a coverage of 30× for tumors and match normal tissues. Raw data from next‐generation sequencing was filtered to remove low‐quality reads and adaptor sequences by fastp (version 0.21.0) [13]. Reads were further aligned to the reference human genome (hg19) utilizing BWA aligner software (version 0.7.10) [14]. BAM files were then sort and mark duplicates by samtools (version 1.1) [15] and GATK (version 4.1.2.0) [16].

### 2.3 Single Nucleotide Variants and Small Insertion/Deletion Detection

SNVs and small indels were called by MuTect2 [17]. Germline mutations were called using GATK software (version 4.1.2.0) and an *in‐house* script [16]. For quality control, somatic mutations were identified if they met the following criteria: (1) present in < 1% of the population in the 1000 Genomes Project (https://www.internationalgenome.org/), the Exome Aggregation Consortium (ExAC), and the Genome Aggregation Database (gnomAD) (https://gnomad.broadinstitute.org); (2) not present in paired germline DNA from normal tissues; and (3) detected in at least two high‐quality reads containing the particular base, where high‐quality reads were defined as a Phred score ≥ 30, mapping quality ≥ 30, and without paired‐end reads bias.

### 2.4 Somatic SNV Identification and Tumor Purity Estimation

A purity > 0.2 was adopted as a filter for samples subjected to CNV analysis. The CNVs was identified by GISTIC 2.0 to analyze the significantly altered copy number of the segments in samples, and a *q*‐value of 0.10 was set as the threshold of significance [18]. Segmentation files obtained from GATK (version 4.1.2.0) were used as the inputs. CNVs gain was defined as segments with copy number/ploidy ≥ log2 (2.5/2), while CNVs loss was segmented with copy number/ploidy < log2 (1.5/2). The tumor purity for each sample was estimated by ABSOLUTE (v1.2) [19].

### 2.5 Identification of Significantly Mutated Genes With Driver Potential

Maftools (version 2.2.10) was used to identify the highly mutated genes and visualize their frequencies in all samples [20]. Non‐silent (deleterious) mutations include nonsense mutations, nonstop mutations, splice‐site mutations, translation start sites, and in‐frame/frame‐shift small indels. Putative driver genes were annotated by the 411 canonical cancer drivers altered in coding sequences listed in the NCG7.0 database [21].

### 2.6 Tumor Mutational Burden

The number of somatic coding nonsynonymous variants (in‐frame/frame‐shift indels, missense mutations, nonsense mutations, non‐stop mutations, splice‐site mutations, and translation start sites) per megabase (Muts/Mb) of the examined genome was calculated as the TMB. TMB data of 26 cancer types from the TCGA database were extracted from Maftools (version 2.2.10).

### 2.7 Published Data Obtained

The genetic data and clinical information of three gastric adenocarcinoma cohorts from previously profiled populations in China (90), Japan (577) and TCGA (438) were collected from the International Cancer Genome Consortium (ICGC). We selected exonic sequencing data from each cohort for comparison analysis in this study (Table S1). Sample DO38284 in TCGA cohort was excluded because of data abnormality. All downloaded data were subjected to a uniform *in‐house* filter pipeline to improve the unity of data from different origins and used for further analysis.

### 2.8 Statistical Analysis

Two‐tailed wilcoxon rank‐sum test was used to analyze differences among GA‐FG and other three gastric cancer cohorts. The statistical significance of KEGG presented pathways in GA‐FG samples using the mutated driver gene list was also calculated by two‐tailed wilcoxon rank‐sum test. All statistical analyses were performed with R v4.0.0 software. Statistical significance was defined as a two‐sided *p* < 0.05.

## 3 Results

### 3.1 Clinical Characteristics of GA‐FG Patients

In total, 21 patients (10 male, 11 female) diagnosed by endoscopy and histology examinations as GA‐FG without priorly receiving preoperative chemotherapy or radiotherapy were included in this study (Table 1 and Table S2). The mean age at diagnosis was 59.9 years (range: 38–81 years), and 13 patients (61.9%) were beyond 60 years. Most patients (*n* = 16, 76.2%) were endoscopically examined as a single lesion, whereas the rest of five cases (23.8%) had two lesions. All lesions were found in the upper stomach, none of them were detected in gastric antrum or pylorus. GA‐FG were resected by endoscopic surgery, including ESD and EMR. Histologically, an infiltrative growth pattern with submucosa invasion was observed in all cases, in accordance with the criteria of the WHO classification [1]. Lymphatic metastasis was absent in all patients. After the follow‐up period of 36 months, all patients were still alive. Representative endoscopic and histological images are shown in Figure S1.

**TABLE 1:** GA‐FG patients' clinical characteristics.

Number of cases	21
Sex	
Female	11
Male	10
Age at diagnosis	
30–39	2
40–49	3
50–59	3
60–69	8
70–79	3
> 80	1
Tobacco status	
Current smoker	1
Ex‐smoker	0
Never	20
Alcohol status	
Current drinker	0
Ex‐drinker	0
Never	21
*H. pylori* infection	
Positive	3
Negative	10
Not detected	8
Paris classification	
0‐IIa	16
0‐IIa‐IIb	1
0‐IIa‐IIc	3
0‐IIc	1
Lymphatic metastasis	
Positive	0
Negative	21
Three‐year survival rate	
Yes	21
No	0
Recurrence interval	
24 months	1
6 months	1
4 months	1
No	18

### 3.2 Genetic Architecture of GA‐FG

**FIGURE 1:**
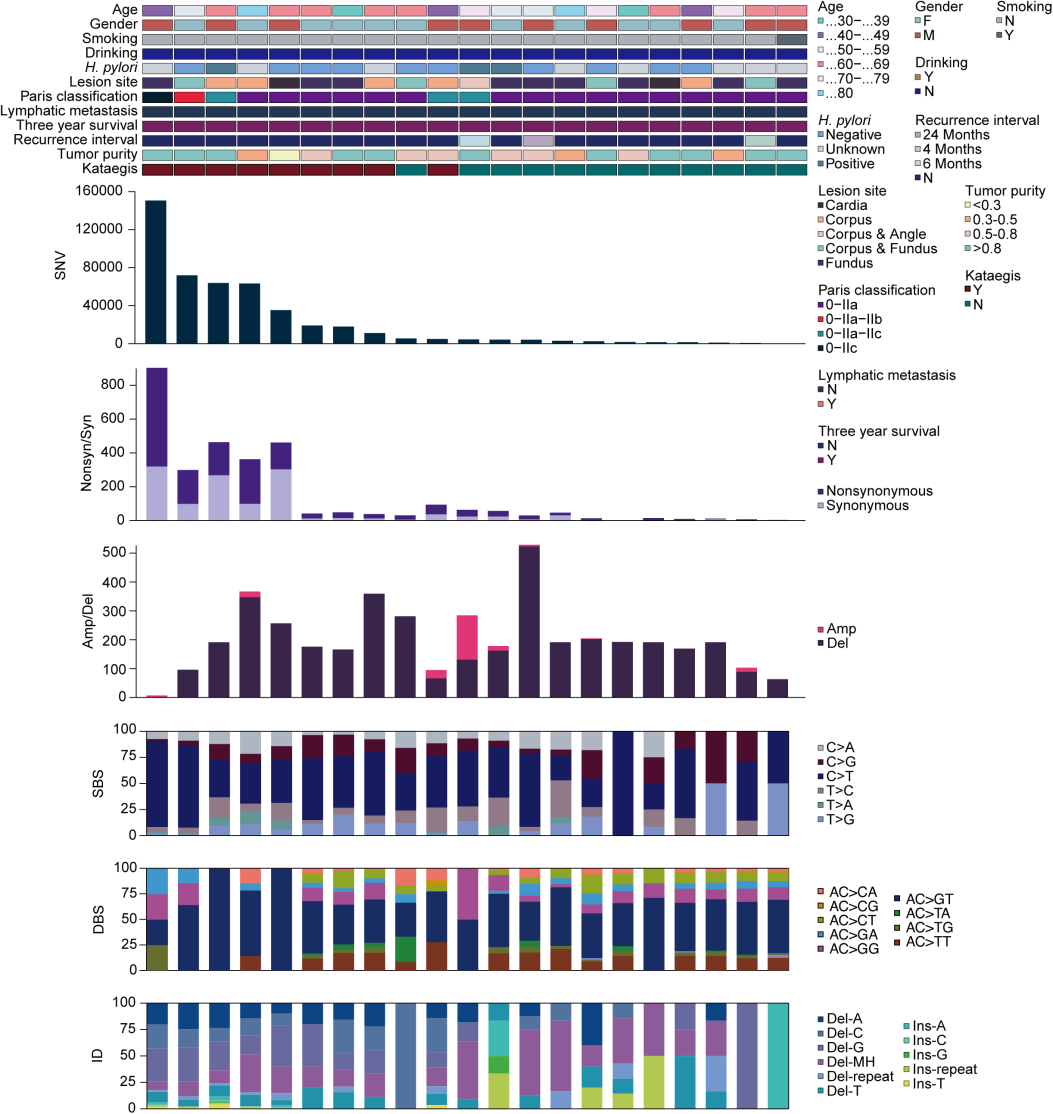
Mutational landscape of the GA‐FG cohort. Each column represents an individual patient (*n* = 21). The top panel shows clinical characteristics, including age, gender, tobacco smoking, alcohol drinking, *H. pylori* infection, neoplastic lesion site, Paris classification, lymphatic metastasis, 3‐year follow‐up, recurrence interval, tumor purity and “kataegis”, as per the color key. Each subsequent panel displays a specific molecular profile: Number of SNV in the whole genome; synonymous and nonsynonymous mutations in the coding regions; amplification and deletion sizes per sample (Mb); and the fractions of SBS, DBS and ID in the genome.

We performed WGS on 21 tumor‐normal paired FFPE samples to a mean depth of 29× to identify somatic mutations (Tables S3 and S4). Sequence coverage exceeding 10× was 88.9% for tumors and 89.0% for normal tissues. We detected a total of 606,124 somatic mutations including SNVs and small insertions and deletions (Indels) (Figure 1), among which missense mutations predominated in all mutation types (Figure S2A). A median of 4541 SNVs (range: 408–150,734) and 965 Indels (range: 116–35,206) for each patient were identified, of which 3146 (0.52% of total mutations) candidate somatic mutations occurred within coding regions were predicted to link to 1698 genes. The number of SNVs per tumor patient genome varied greatly (median: 4541, range: 408–150,734). In the coding areas, we detected a median of 40 SNVs per sample (range: 2–877), among which the median number of nonsynonymous SNVs is 25 per patient (range: 2–567), which is significantly lower than in traditional gastric cancer by other published studies [22, 23].

To further explore the GA‐FG‐related molecular events, we analyzed genomic copy number alterations (CNAs) by GISTIC (Figure 1). Deletion dominated in most samples, with a median size of 184.55 megabase (Mb) per case (range: 0–504.33 Mb). Correspondingly, we identified that 16p11.2 (*q* = 0.018, two‐sided Wilcoxon rank‐sum test, Table S5) containing several *TP53*‐inducible genes such as *TP53TG3*, *TP53TG3C* and *TP53TG3B* was the only chromosome area with gain copy numbers. 1p36.21 (*q* = 0.027, two‐sided Wilcoxon rank‐sum test) and 19p13.3 (*q* = 1.26E‐05, two‐sided Wilcoxon rank‐sum test) were the most significantly deleted chromosome regions (Figure S2E and Table S6), a pattern also observed in the intestinal‐type of conventional gastric cancers [23]. KEGG enrichment analysis revealed that genes deleted in 19p13.3 arm (*APC2*, *FGF22*, *MAP2K2*, *GADD45B*, *SHC2*, et al.) were converged on gastric cancer, cAMP, Ras and FoxO signaling pathways (Figure S2F), implying that loss of 19p13.3 likely contributed to the carcinogenesis of GA‐FG.

In examining the base substitution spectrum, we observed a markedly more prevalent distribution of transitions than transversions across the whole genome (Figure S2D), and yielded a total of 470,040 somatic single‐base substitution (SBS, median: 4540, range: 408–150,584), with a high transition rate of C > T mutation (50.4%) among the 6 base conversion categories (Figure 1 and Figure S2B,C). For doublet‐base substitutions (DBS) and small insertion‐and‐deletion (ID), the AC > GT (53.7%) and deletions with microhomology (Del‐MH, 25.1%) mutations constituted the largest proportions in all samples (Figure 1).

Hypermutated genomic regions, known as “Kataegis”, were identified in 42.8% of samples, with an average of 50 events per case (range: 1–165). Rainfall plot for patient GA9908 was shown in Figure S2G. Hypermutated regions were found in chromosome 3, 5, 9, 18, 20 and 23.

### 3.3 Genomic Disparities Between GA‐FG and Conventional Gastric Cancer

**FIGURE 2:**
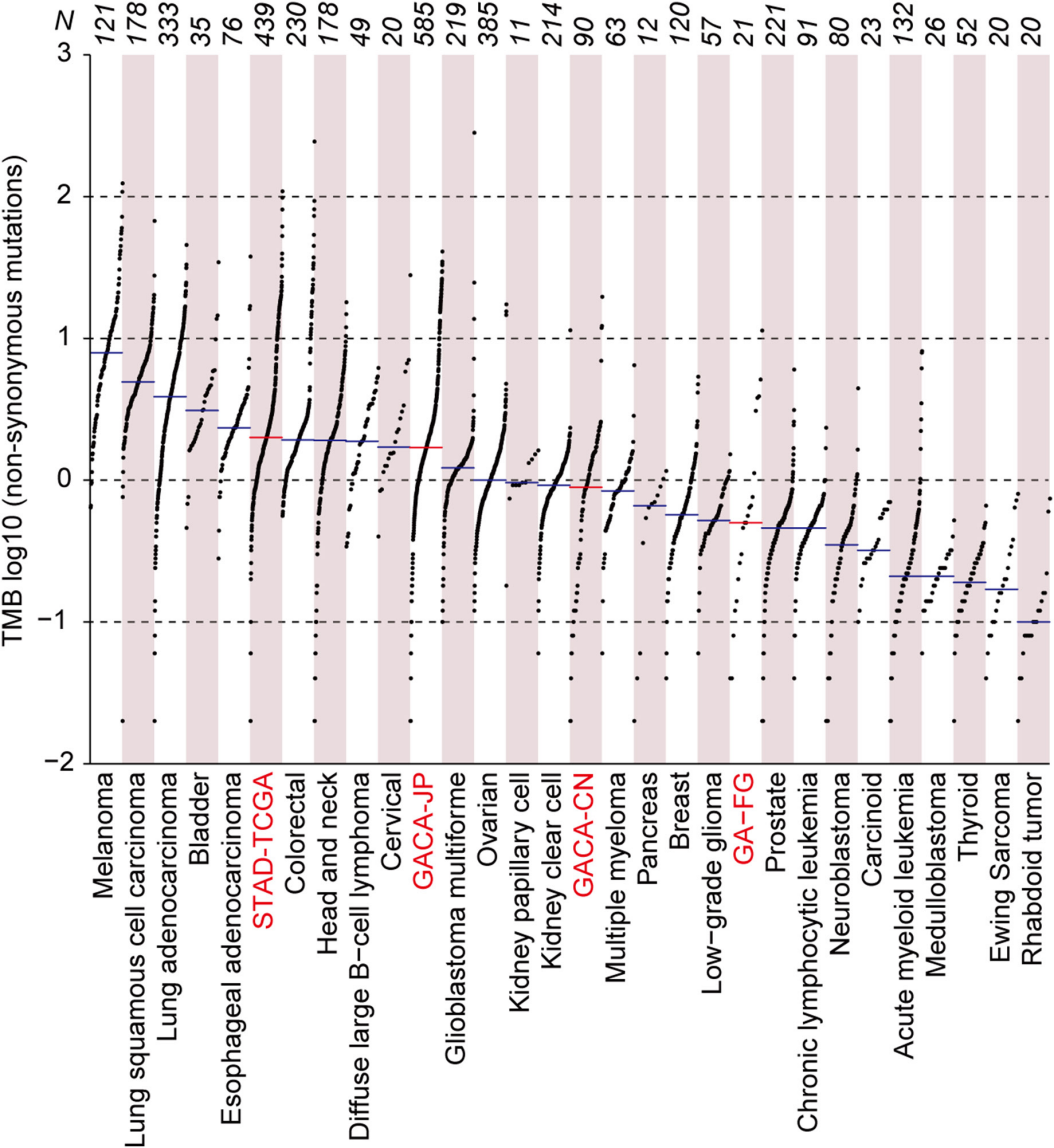
Genomic disparities between GA‐FG and traditional gastric cancer. TMB across GA‐FG, 3 cohorts of traditional gastric cancers and other 26 cancer types from the TCGA study. Each dot represents a sample, the total sample numbers for each type are shown at the top. The red horizontal lines are the median numbers of mutations per megabase (log_10_). GA‐FG and 3 gastric cancer cohorts were marked as red color.

We next sought to systematically characterize the genetic discrepancies between GA‐FG and conventional gastric cancer by incorporating International Cancer Genome Consortium (ICGC) datasets from 1105 patients with traditional gastric adenocarcinomas, representing three cohorts from previously profiled populations in China (90), Japan (577) and TCGA (438), respectively (Methods, Table S1). We firstly measured the degree of intra‐tumor heterogeneity in GA‐FG and conventional gastric cancer patients by calculating the mutant‐allele tumor heterogeneity (MATH) scores for each patient using R package maftools. Here we detected similar MATH scores for GA‐FG and conventional gastric cancer patients (Figure S3A). Non‐synonymous TMB was then calculated, showing a great variation across the GA‐FG cohort (Figure S3B), with a median mutation burden rate of 0.5 per Mb (range: 0.04–11.34). GA‐FG cohort displayed a 1.78‐fold lower of TMB than gastric cancer patients in Chinese cohort (median: 0.89, range: 0.02–19.62, *p* = 0.41, two‐sided Wilcoxon rank‐sum test, Figure 2A and Figure S3C), although statistical significance was not observed between these two cohorts. However, TMB in GA‐FG patients was more than 3‐fold lower than in Japanese cohort (median: 1.7, range: 0.02–40.92, *p* = 4.4 × 10^−16^, two‐sided Wilcoxon rank‐sum test), and 4‐fold lower than in TCGA cohort (median: 2, range: 0.02–108.86, *p* = 1.1 × 10^−5^, two‐sided Wilcoxon rank‐sum test), indicating low mutation accumulation in GA‐FG patients.

TCGA Research Network has identified four distinct molecular subtypes of conventional gastric cancer based on their molecular characteristics, including tumors positive for (1) Epstein–Barr virus (EBV), (2) microsatellite instability (MSI), (3) chromosomal instability (CIN), and (4) genome stability (GS) [24]. Meanwhile, The Asian Cancer Research Group (ACRG) has also proposed (1) MSI, (2) microsatellite stability (MSS)/epithelial‐mesenchymal transition (EMT), (3) MSS/TP53^+^, and (4) MSS/TP53^−^ in gastric cancers [25]. To compare the mutational discrepancies between GA‐FG and other subtypes of gastric cancer, we calculated the non‐synonymous TMB in these samples. We revealed that, apart from the two MSI subgroups characterized by the TCGA and ACRG, respectively, GA‐FG samples showed similar mutation burden rate with their counterparts in conventional gastric cancers (Figure S3D,E). By contrast, TMB in MSI subtypes were significantly larger than those from other subtypes and GA‐FG samples (Figure S3D,E).

Previous evidence indicated that GA‐FG is a novel type of gastric cancer that is not related to *H. pylori* infection [8]. To investigate the mutation accumulation in GA‐FG patients with or without *H. pylori* infection, we calculated the non‐synonymous TMB in these patients, revealing that *H. pylori*‐positive GA‐FG patients tended to harbor higher TMB scores compared to their *H. pylori*‐negative counterparts, although no statistical significance was detected in these subjects (Figure S3F).

### 3.4 Significantly Mutated Genes (SMGs) With Driver Potential in GA‐FG and Gastric Adenocarcinoma

**FIGURE 3:**
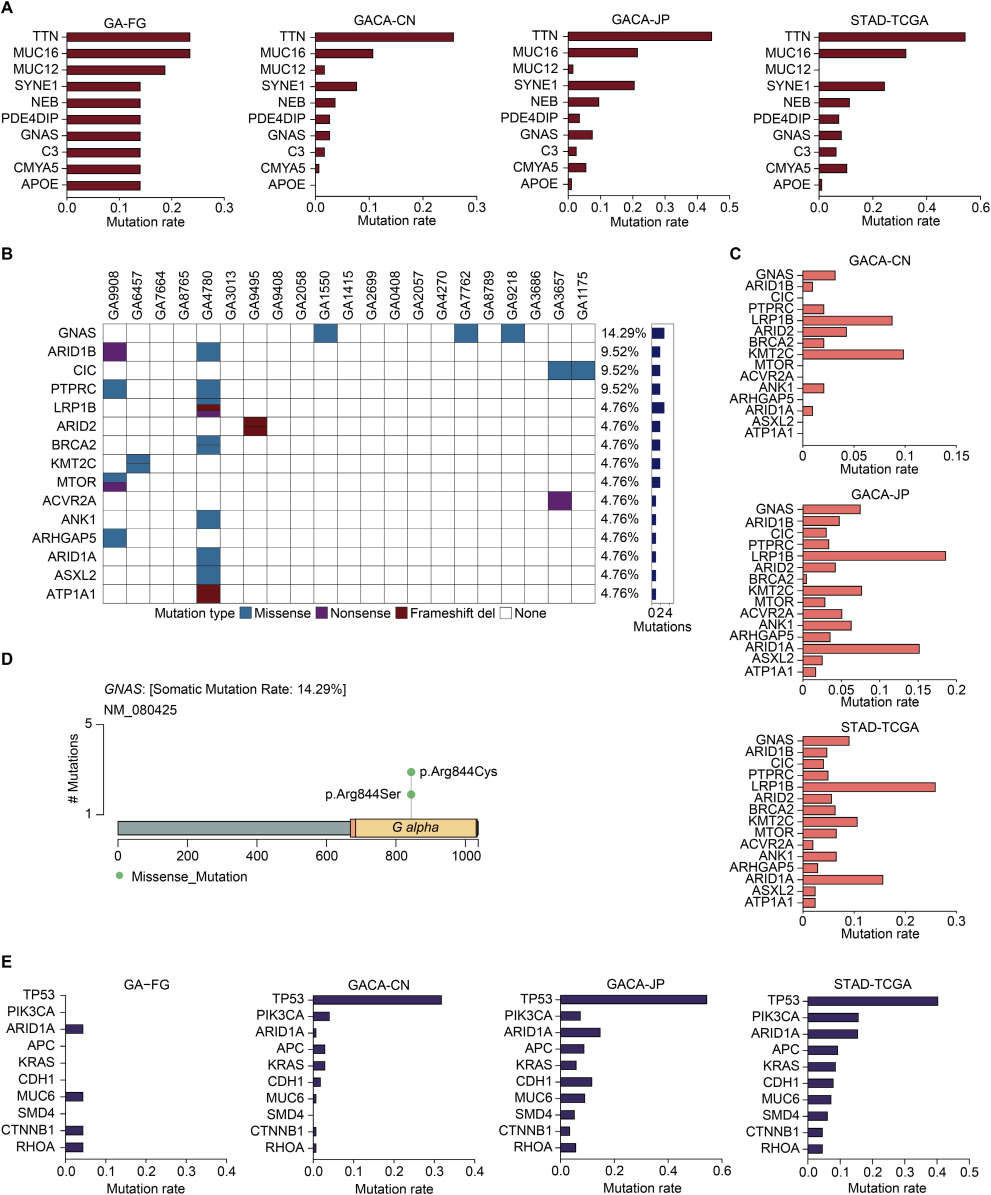
SMGs with driver potential in GA‐FG and 3 gastric cancer cohorts. (A) The top 10 SMGs in GA‐FG samples were shown. Nonsynonymous mutation rates of these SMGs in GA‐FG were compared with 3 gastric cancer cohorts. (B) The top 15 recurrently mutated driver genes identified in GA‐FG cohort. Each column represents an individual patient. Upper, patients ID; Lower, nonsynonymous mutation types; Right, mutation numbers. (C) Mutational frequencies of the top 15 driver genes in GA‐FG samples were identified in other 3 gastric cancer cohorts. (D) Lollipop plot displaying mutation distribution and protein domain for *GNAS* in GA‐FG samples. Somatic mutation rate and transcript names are indicated by plot title and subtitle, respectively. (E) Mutational frequencies of the top 10 driver genes in TCGA cohort were identified in GA‐FG and other 2 gastric cancer populations.

A previous study using the Ion Ampliseq Cancer Hotspot Panel v2 reported frequent oncogenic mutations including *GNAS*, *KRAS*, *PIK3CA* genes in GA‐FG samples [8]. In order to gain comprehensive insights into the genetic alterations that may be related to carcinogenesis, we applied Maftools package to identify SMGs by calculating the non‐silent somatic mutations [20], including missense, nonsense, splice‐site, and frame‐shift mutations, in GA‐FG cohort. Top 10 SMGs (*FDR* < 0.1) were summarized in Figure 3A, showing that *TTN* and *MUC16* have the highest mutation frequencies (23.8%), followed by *MUC12* (19.1%), *SYNE1* (14.3%), *NEB* (14.3%), *PDE4DIP* (14.3%), *GNAS* (14.3%), *CMYA5* (14.3%) and *APOE* (14.3%). By integrating these data with populations from conventional gastric cancer cohorts, we revealed that *TTN* and *MUC16* also mutated frequently in CN (*TTN*: 26%, *MUC16*: 11%), JP (*TTN*: 45%, *MUC16*: 22%) and TCGA (*TTN*: 55%, *MUC16*: 33%) populations. Meanwhile, we observed relatively low mutation rates of *MUC12* and *APOE* in gastric cancer patients, which is different from GA‐FG disease. We further determined the mutation frequencies of these SMGs in gastric cancer subtypes categorized by TCGA. We revealed that, apart from *MUC12* and *APOE*, other SMGs also showed high mutation frequencies in the four subgroups, especially in the MSI subgroup (Figure S4A). Probably due to the different sequencing platform in ACRG samples, the non‐silent somatic mutations of many SMGs were not detected in the ACRG cohort (Figure S4B).

We next selected canonical driver genes annotated by NCG7.0 database from the SMGs list for further analysis [21]. Top 15 mutated driver genes were shown in Figure 3B, showing a relatively moderate mutation prevalence in GA‐FG populations. Among them, *PTPRC* (9.52%), *ARID1A* (4.76%) and *ACVR2A* (4.76%) were previously reported as significantly mutated driver genes in common gastric adenocarcinomas cohorts (Figure 3C). Other 12 unreported drivers, including *GNAS* (14.29%), *ARID1B* (9.52%), *CIC* (9.52%), *LRP1B* (4.76%), *ARID2* (4.76%), *BRCA2* (4.76%), *KMT2C* (4.76%), *MTOR* (4.76%), *ANK1* (4.76%), *ARHGAP5* (4.76%), *ASXL2* (4.76%), *ATP1A1* (4.76%) indicated unique mutational processes in the carcinogenesis of GA‐FG. *GNAS* (located in 20q13.32), ranking the most significantly mutated driver gene, harbored 3 missense mutations in the same position, including p.Arg844Cys and p.Arg844Ser (Figure 3D). We detected a similar mutation rate of *GNAS* in common gastric cancer populations. Other key drivers such as *ARID1B* (located in 6q25.3) and *CIC* (located in 19q13.2) were also mutated moderately in common gastric cancer cohorts (Figure 3C). By calculating the mutational frequencies of these driver genes in subtypes from the TCGA cohort, we similarly detected high mutation rates of *GNAS* and *ARID1B* in the EBV and MSI subgroups, but not in other subtypes (Figure S4C).

We further calculated the mutational frequencies of several well‐known driver genes, including *TP53*, *PIK3CA*, *APC*, *KRAS*, *CDH1* and *SMD4* [24], in the GA‐FG cohort, and in the subtypes characterized by TCGA and ACRG (Figure 3E and Figure S4E,F). All of them have been established as key players in the carcinogenesis of gastric adenocarcinoma. Surprisingly, compared to the high mutation rates in common gastric cancer cohorts, we didn't detect any non‐silent mutations of these genes in GA‐FG patients. Nonsynonymous mutation of other key drivers such as *MUC6*, *CTNNB1* and *RHOA* were just found in one case. Thus, our SMGs analysis uncovered distinct genetic mutation alterations in GA‐FG samples, with the absent involvement of previously established driver genes in the tumorigenesis of GA‐FG.

We next profiled SMGs in GA‐FG patients with or without *H. pylori* infection, and observed that SMGs such as *MUC16*, *MUC12* and *TTN* were identified in patients regardless of *H. pylori* status (Figure S5A). Interestingly, non‐silent *GNAS* mutation was solely identified in *H. pylori*‐negative GA‐FG patients, suggesting that *GNAS* mutation was not correlated with *H. pylori* infection.

### 3.5 Oncogenic Signaling Pathways in GA‐FG and Traditional Gastric Cancer

**FIGURE 4:**
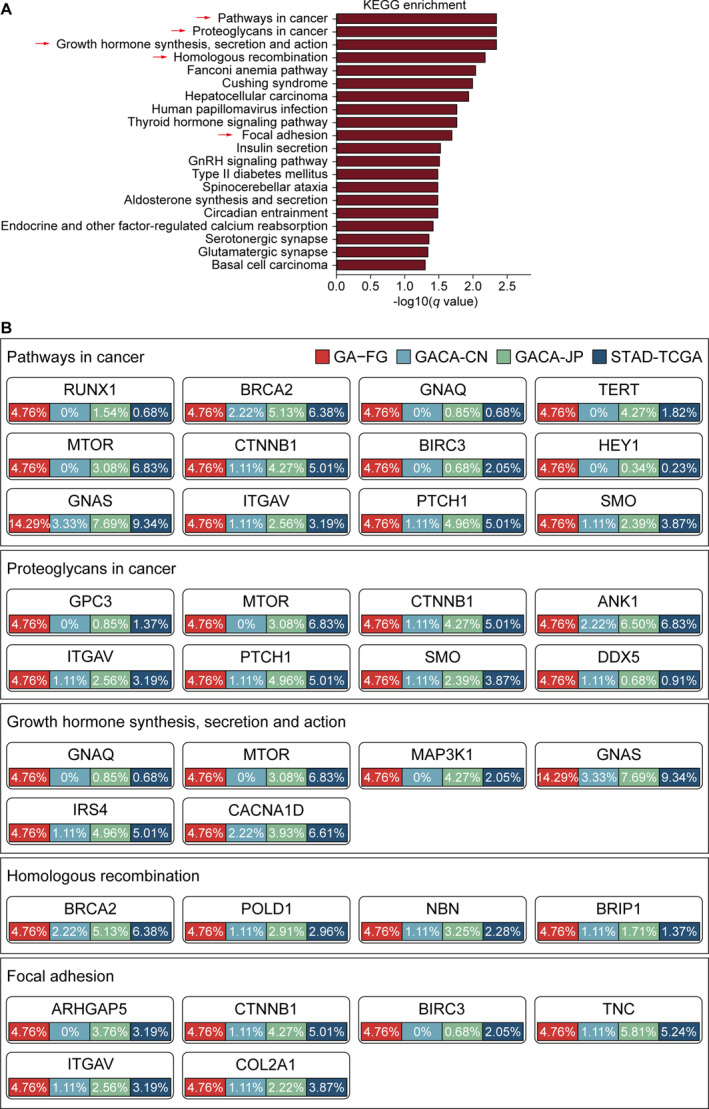
Oncogenic signaling pathways in GA‐FG and traditional gastric cancer. (A) Bar plots of the top 20 KEGG pathways based on the −log_10_ (*q*‐value) that biologically enriched from GA‐FG‐specific driver genes. (B) Mutational frequencies of driver genes involved in the five indicated oncogenic pathways were calculated in GA‐FG and other 3 gastric cancer cohorts.

To further understand the biological function of significantly mutated driver genes, we searched for over‐represented pathways in KEGG database by using the list of non‐silently mutated driver genes. Top 20 altered signaling pathways (*p* < 0.01, two‐sided Wilcoxon rank‐sum test) were presented in Figure 4A. Focal adhesions pathway (*p* = 0.001, *q* = 0.021, two‐sided Wilcoxon rank‐sum test) has also been revealed to be involved in common gastric carcinogenesis by other published studies [23], indicating that perturbed cell adhesion and mobility might also affect the progression and invasion of GA‐FG. Other top enriched pathways, including proteoglycans in cancer (*p* = 2.9 × 10^−5^, *q* = 0.0046, two‐sided Wilcoxon rank‐sum test), growth hormone synthesis, secretion, and action (*p* = 8.1 × 10^−5^, *q* = 0.0046, two‐sided Wilcoxon rank‐sum test) and homologous recombination (*p* = 1.58 × 10^−4^, *q* = 0.0068, two‐sided Wilcoxon rank‐sum test) were significantly presented in GA‐FG populations. We next sought to identify pathways enriched in GA‐FG patients regardless of the *H. pylori* status, and observed that several metabolism‐related processes, including cholesterol, carbon, phenylalanine, and vitamin metabolism, were over‐represented in these patients (Figure S5B).

By mapping significantly mutated driver genes to these signaling pathways (Figure 4B), we observed that proteoglycans in cancer pathway carried eight genes with non‐silent mutations, including *GPC3*, *MTOR*, *CTNNB1*, *ANK1*, *ITGAV*, *PTCH1*, *SMO* and *DDX5*, which also exhibited moderate to high mutation prevalence in gastric cancer cohorts. Focal adhesion pathway carried six driver genes with nonsynonymous mutations such as *CTNNB1*, *ITGAV* and *COL2A1*. In growth hormone synthesis, secretion, and action pathway, we observed mutations in *GNAS*, *MTOR*, *GNAQ*, *MAP3K1*, *IRS4* and *CACNA1D*, supporting the pivotal role of *GNAS* in maintaining the production of several hormones that help regulate the activity of endocrine glands such as the thyroid, pituitary glands, and adrenal glands [26].

## 4 Discussion

To date, apart from a handful of cases reports and immunohistochemistry studies [8, 27], the genomic features of GA‐FG remain largely unknown. In the present study, we have shown for the first time, to our knowledge, the systematical characterization of genetic alterations accumulating in GA‐FG with single‐nucleotide resolution. By comprehensively exploring the somatic SNVs and CNVs, as well as predicting potential significantly mutated driver genes and mutational signatures, we herein uncovered the underlying mutagenic processes of such neoplasm. Moreover, comparative analysis of GA‐FG samples and conventional gastric adenocarcinoma populations enabled us to reveal basic genetic characteristics both common to gastric cancers and unique to GA‐FG disease.

In this study, we identified extensively genetic evidence in GA‐FG, including (1) low mutational burden; (2) small numbers of non‐silent somatic SNVs; (3) relatively low mutational frequencies of SMGs with driver potential; (4) lack of formerly established driver gene mutations such as *TP53*, *KRAS* and *CDH1*. TMB refers to the number of certain mutations carried by tumor cells, usually a footprint of powerful carcinogen exposure such as ultraviolet light and alcohol drinking [28, 29]. We herein uncovered a markedly low nonsynonymous mutation accumulating during the progression of GA‐FG as compared with those gastric cancer cohorts. As TMB was recently cited by physicians as a predictor for immune checkpoint inhibitor (ICI) therapies [29], low mutational burden may imply an inefficiency of GA‐FG patients to respond to these treatments. In line with these findings, we further detected a relatively low to moderate mutation rates of significantly mutated driver genes in GA‐FG populations, substantiating limited contribution of “hazardous” mutations to the pathogenesis of GA‐FG disease.

By mapping the significantly mutated genes with driver potential, we revealed a number of SMGs both mutated frequently in GA‐FG and gastric cancer populations. *TP53* is a key cellular stress sensor regulating a bunch of cell growth arrest genes. Mutation in *TP53* abrogates the ability to detect DNA damage, thus leading to the aberrant cell proliferation [30]. Likewise, *APC* as a well‐known tumor suppressor gene involved in a variety of cellular processes such as cell migration, adhesion, and chromosome segregation. Mutation of *APC* is an early event for > 80% of sporadic colorectal cancers (CRC) [31]. Inactivation of these “caretaker” genes contributes to genome instability, which are pivotal cellular processes for the tumorigenesis of gastric cancer. However, in the present study, we observed an absence of multiple driver gene mutations in GA‐FG cohort, including *TP53*, *PIK3CA*, *APC*, *KRAS*, *CDH1*, and *SMD4*.

Instead, we observed that *GNAS* is the driver gene with the highest mutation rate in GA‐FG cohort, which is consistent with several previous publications [8, 27]. Previous studies have reported *GNAS* to be mutated in intestinal‐phenotype of gastric adenocarcinomas and involved in pancreatic tumorigenesis [32, 33]. A recent evidence showed recurrent *GNAS* mutation in a significant proportion of gastric foveolar metaplasia and gastric heterotopia [34], suggesting that such genetic alteration may contribute to the proliferation of metaplastic epithelium, which is an early predictor for the development of gastric cancer. Of noted, *GNAS* mutation is possible to be linked with deep submucosal invasion and increased tumor size [6], highlighting an underlying oncogenic role in the progression and invasion of GA‐FG and conventional gastric adenocarcinoma. Similarly, we observed that *MUC12*, a transmembrane mucin produced by epithelial cells with a key role in mucosal protection and function [35], was exclusively mutated in GA‐FG populations. *MUC12* is not prognostic in gastric cancer, but notably associated with poor prognosis of adrenocortical carcinoma and glioblastoma [36]. Although functional validation of *GNAS* and other identified SMGs are still needed in geographically separated cohorts with well‐phenotyped patients, our results suggest that detection the molecular alteration of *GNAS* and *MUC12* might be help in the diagnosis of GA‐FG in the future.

Currently, the majority of GA‐FG patients were treated endoscopically [3]. We discovered that homologous recombination was perturbed in the GA‐FG populations, which is also presented in a genome‐wide study containing over 470 gastric cancer samples, revealing that 7%–12% of gastric cancer patients carried that signature [37]. Breast, ovarian and pancreatic cancer patients with defective homologous recombination caused by *BRCA1* and/or *BRCA2* mutations always benefit from platinum therapy or poly (adenosine diphosphate ribose) polymerase (PARP) inhibitors (PARPi), due to their incapability of effectively repairing the double‐strand breaks [38, 39], implying that platinum and PARPi might be promising therapies for GA‐FG patient harboring *BRCA2* mutation.

In summary, our study depicts a genomic landscape highlighting the multidimensional perturbations in GA‐FG genome. Comparative analysis of GA‐FG samples with traditional gastric cancer cohorts revealed unique mutational signatures in GA‐FG populations, as well as common genetic alterations shared by conventional gastric adenocarcinoma. These findings will undoubtedly advance our current knowledge and provide further insights for novel diagnostic and therapeutic strategies for patients with this disease.

## Author Contributions

**Hu Wei:** data curation (lead), methodology (lead). **Ze Min Chen:** data curation (equal), methodology (equal). **Xiu Fen Xue:** methodology (equal), resources (lead). **Li Xia Xi:** data curation (supporting), methodology (supporting). **Gen Hua Yang:** data curation (supporting), resources (supporting). **Zhi Yong Zhai:** data curation (supporting), resources (supporting). **Zhao Yu Huang:** data curation (supporting), resources (supporting). **Ping Zhou:** resources (supporting). **Chong Ju Bao:** resources (supporting). **Li Juan You:** data curation (supporting), resources (supporting). **Mei Ping Ou Yang:** data curation (supporting), resources (supporting). **Gui Li Xia:** data curation (supporting), resources (supporting). **Zhi Yu Zeng:** data curation (supporting), resources (supporting). **Xiao Bing Cui:** project administration (equal), supervision (equal). **Xiao Juan Pei:** project administration (equal), resources (lead). **Wei Gong:** conceptualization (lead), funding acquisition (lead), project administration (lead), resources (lead), supervision (lead).

## Ethics Statement

This study was approved by the Ethic Committee of Southern Medical University (NYSZYYEC20190013).

## Consent

The authors have nothing to report.

## Conflicts of Interest

The authors declare no conflicts of interest.

## Supporting information


**Figure S1.** Representative endoscopic and immunohistochemical images for each GA‐FG patient were shown. Immunohistochemical images were displayed at different resolutions of 10× and 40×, respectively.

**Figure S2.** Genetic architecture of GA‐FG. (A) Bar plots of the number of detailed mutation classifications. (B–D) Fractions of the types of SNVs, including six types of SNVs (B, C) and transitions vs transversions (D). (E) Genomic regions with significant recurrent somatic CNAs. Genes with a copy number loss or gain in GA‐FG samples were indicated. (F) Bar plots of the top 5 KEGG pathways based on the gene percentages that biologically enriched from genes deleted in chromosome 19p13.3 in GA‐FG samples. *p*‐value for each pathway was calculated by two‐sided Wilcoxon rank‐sum test. (G) Rainfall plot for GA‐FG GA9908 sample. Each point is a mutation color coded according to SNV class. Hypermutated genomic segments identified by the change‐point method are highlighted by black arrowheads.

**Figure S3.** (A) MATH scores were calculated in GA‐FG patients and gastric cancer populations from Japan and TCGA. (B) TMB values in each GA‐FG samples were calculated. (C) Comparison of nonsynonymous TMB values in GA‐FG and three gastric cancer cohorts. (D) Comparison of nonsynonymous TMB values in GA‐FG and four subtypes of gastric cancers defined by TCGA. CIN: chromosomal instability; EBV: Epstein–Barr virus; GS: Genome stability; MSI: Microsatellite instability. (E) Comparison of nonsynonymous TMB values in GA‐FG and four subtypes of gastric cancers defined by ACRG. MSS/EMT: Microsatellite stability (MSS)/epithelial‐mesenchymal transition (EMT). (F) TMB values in GA‐FG patients with or without *H. pylori* infection were analyzed. Each dot represents a sample. *p* values were calculated by two‐sided Wilcoxon rank‐sum test. Boxes represent the IQRs between the first and third quartiles, and the line inside the box represents the median; whiskers represent the lowest or highest values within 1.5 times IQR from the first or third quartiles.

**Figure S4.** (A) The non‐synonymous mutation rates of the top 10 SMGs in GA‐FG samples were shown in the four gastric cancers subtypes in the TCGA cohort. (B) The non‐synonymous mutation rates of the top 10 SMGs in GA‐FG samples were shown in the four gastric cancers subtypes in the ACRG cohort. (C) Mutational frequencies of the top 15 driver genes in GA‐FG samples were shown in the four gastric cancers subtypes in the TCGA cohort. (D) Mutational frequencies of the top 15 driver genes in GA‐FG samples were shown in the four gastric cancers subtypes in the ACRG cohort. (E) Mutational frequencies of the top 10 driver genes in TCGA cohort were shown in the four gastric cancers subtypes in the TCGA cohort. (F) Mutational frequencies of the top 10 driver genes in TCGA cohort were shown in the four gastric cancers subtypes in the ACRG cohort.

**Figure S5.** (A) SMGs in GA‐FG patients with or without *H. pylori* infection were analyzed using the Maftool package. SMGs simultaneously profiled in GA‐FG patients regardless of *H. pylori* status were showed. (B) Signaling pathways in GA‐FG patients with or without *H. pylori* infection were presented based on the gene percentage.

**Table S1.** ICGC samples included in this study.

**Table S2.** GA‐FG patients’ clinical information.

**Table S3.** Summary of whole‐genome sequencing statistics for the GA‐FG cohort.

**Table S4.** Detailed information of whole‐genome sequencing for the GA‐FG cohort.

**Table S5.** Amplification peaks detected by GISTIC2 and genes residing within those peaks.

**Table S6.** Deletion peaks detected by GISTIC2 and genes residing within those peaks.

## Data Availability

The raw sequence data reported in this paper have been deposited in the Genome Sequence Archive (Genomics, Proteomics & Bioinformatics 2021) in National Genomics Data Center (Nucleic Acids Res 2022), China National Center for Bioinformation/Beijing Institute of Genomics, Chinese Academy of Sciences (GSA‐Human: HRA006370) that are publicly accessible at https://ngdc.cncb.ac.cn/gsa‐human. Raw data are available from the corresponding author upon reasonable request.
