# Supplementary figures and images for: Mutational Landscape of Gastric Adenocarcinoma of the Fundic Gland Type Revealed by Whole Genome Sequencing
Source: Cancer Med. 2024 Oct 9;13(19):e70290. doi: 10.1002/cam4.70290 (PMC11462592; doi:10.1002/cam4.70290)

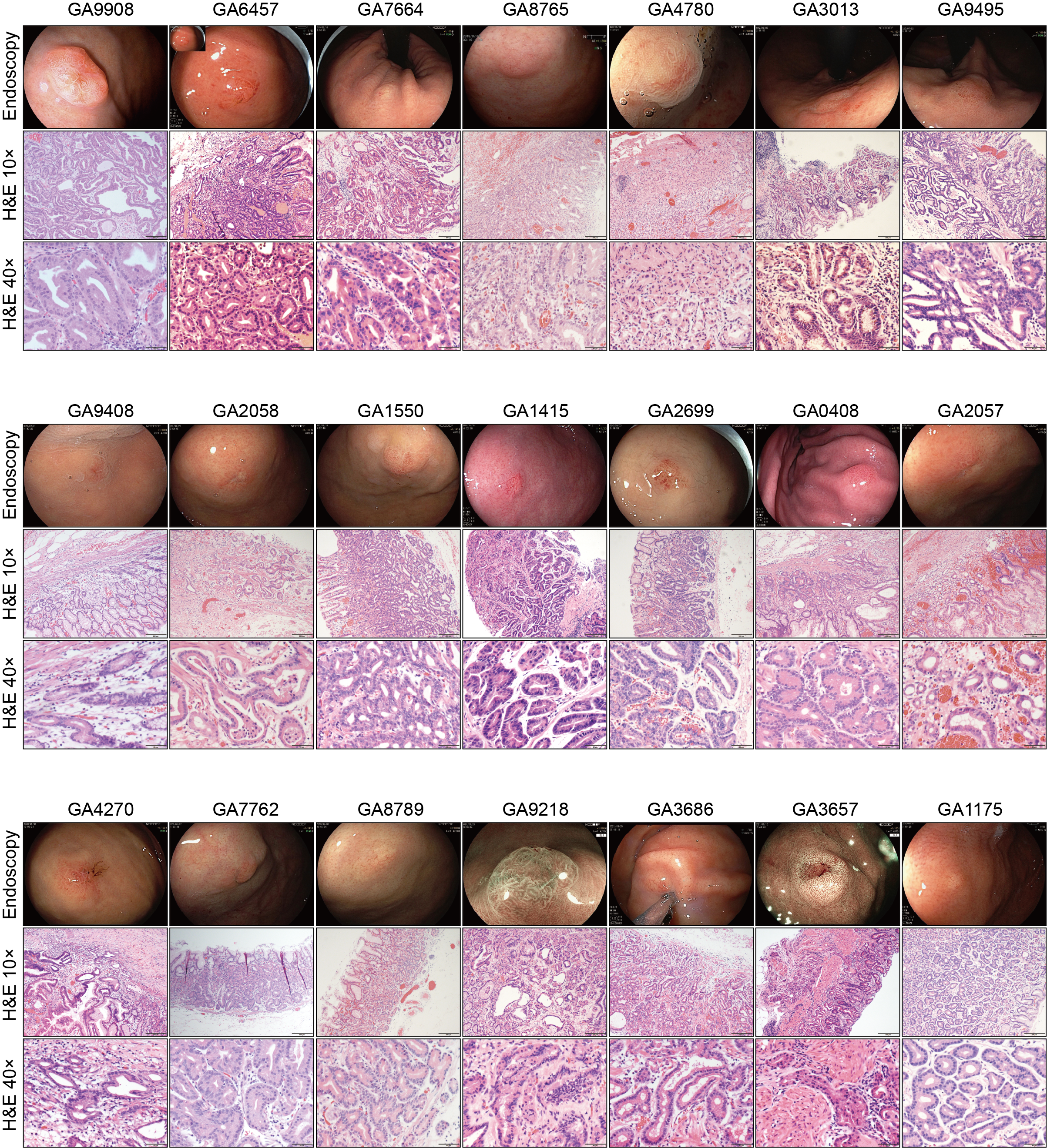

Supplement: Supplementary file 1 — Figure S1. Representative endoscopic and immunohistochemical images for each GA‐FG patient were shown. Immunohistochemical images were displayed at different resolutions of 10× and 40×, respectively. [file CAM4-13-e70290-s002.png]

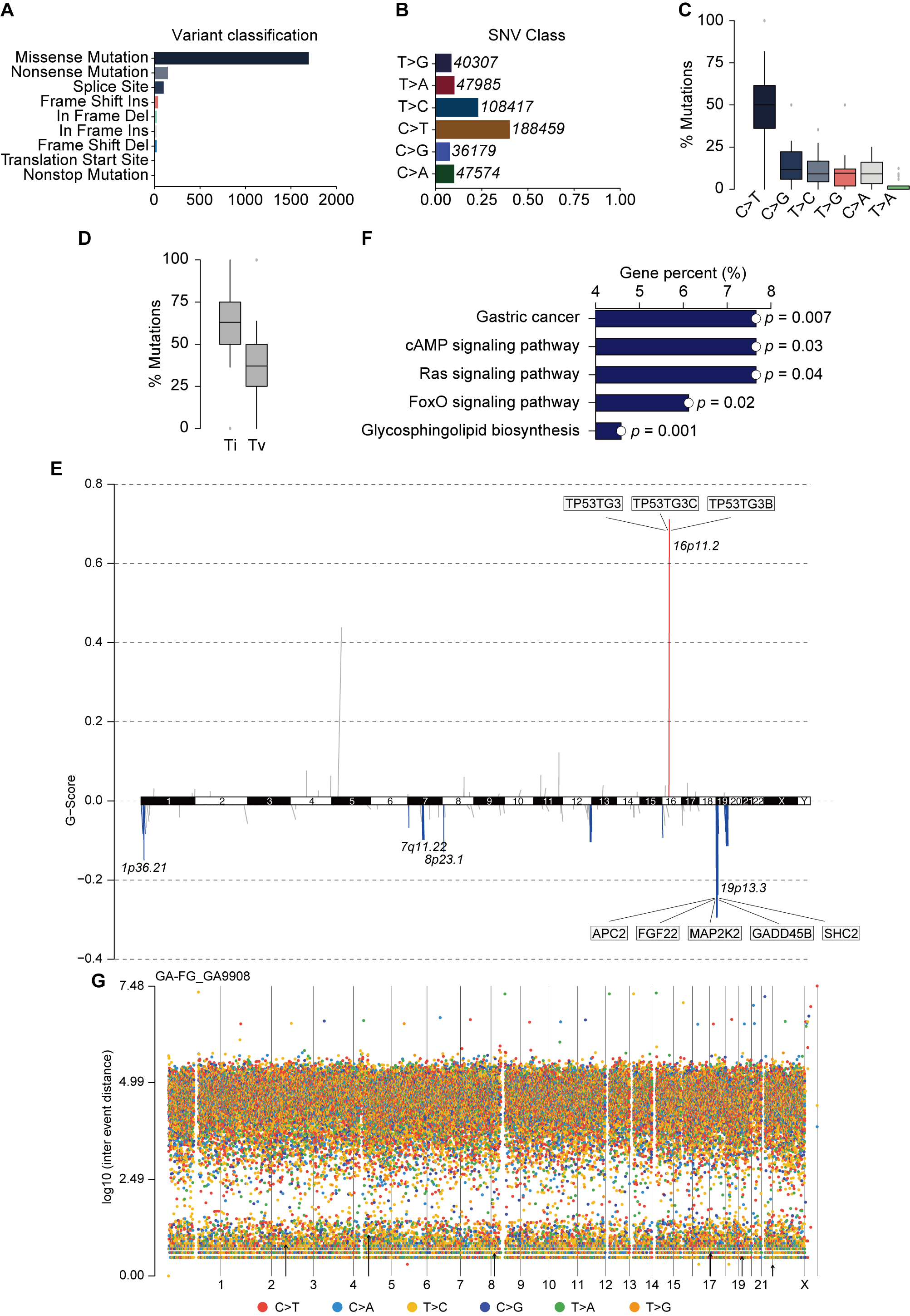

Supplement: Supplementary file 2 — Figure S2. Genetic architecture of GA‐FG. (A) Bar plots of the number of detailed mutation classifications. (B–D) Fractions of the types of SNVs, including six types of SNVs (B, C) and transitions vs transversions (D). (E) Genomic regions with significant recurrent somatic CNAs. Genes with a copy number loss or gain in GA‐FG samples were indicated. (F) Bar plots of the top 5 KEGG pathways based on the gene percentages that biologically enriched from genes deleted in chromosome 19p13.3 in GA‐FG samples. p‐value for each pathway was calculated by two‐sided Wilcoxon rank‐sum test. (G) Rainfall plot for GA‐FG GA9908 sample. Each point is a mutation color coded according to SNV class. Hypermutated genomic segments identified by the change‐point method are highlighted by black arrowheads. [file CAM4-13-e70290-s006.tif]

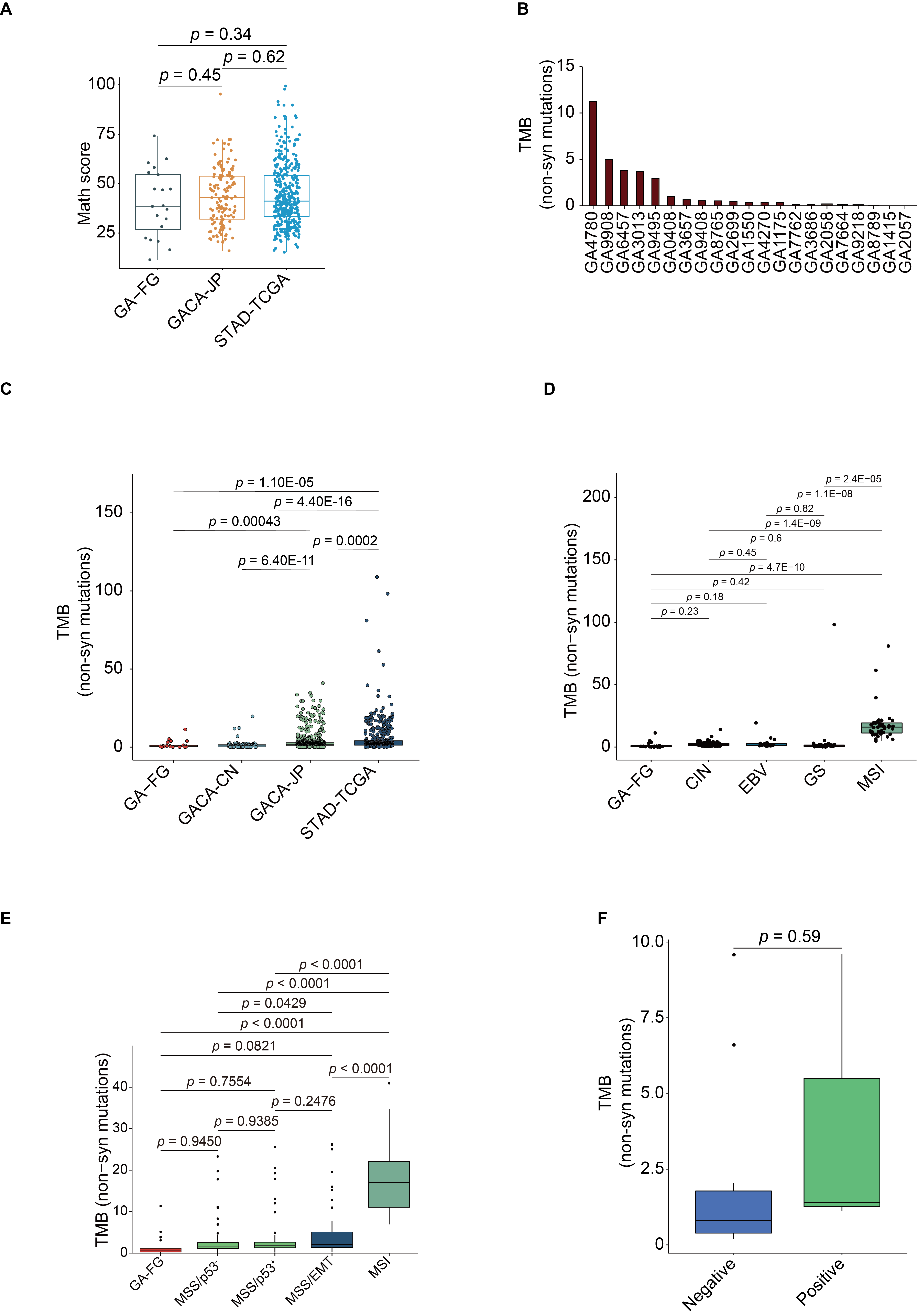

Supplement: Supplementary file 3 — Figure S3. (A) MATH scores were calculated in GA‐FG patients and gastric cancer populations from Japan and TCGA. (B) TMB values in each GA‐FG samples were calculated. (C) Comparison of nonsynonymous TMB values in GA‐FG and three gastric cancer cohorts. (D) Comparison of nonsynonymous TMB values in GA‐FG and four subtypes of gastric cancers defined by TCGA. CIN: chromosomal instability; EBV: Epstein–Barr virus; GS: Genome stability; MSI: Microsatellite instability. (E) Comparison of nonsynonymous TMB values in GA‐FG and four subtypes of gastric cancers defined by ACRG. MSS/EMT: Microsatellite stability (MSS)/epithelial‐mesenchymal transition (EMT). (F) TMB values in GA‐FG patients with or without H. pylori infection were analyzed. Each dot represents a sample. p values were calculated by two‐sided Wilcoxon rank‐sum test. Boxes represent the IQRs between the first and third quartiles, and the line inside the box represents the median; whiskers represent the lowest or highest values within 1.5 times IQR from the first or third quartiles. [file CAM4-13-e70290-s011.png]

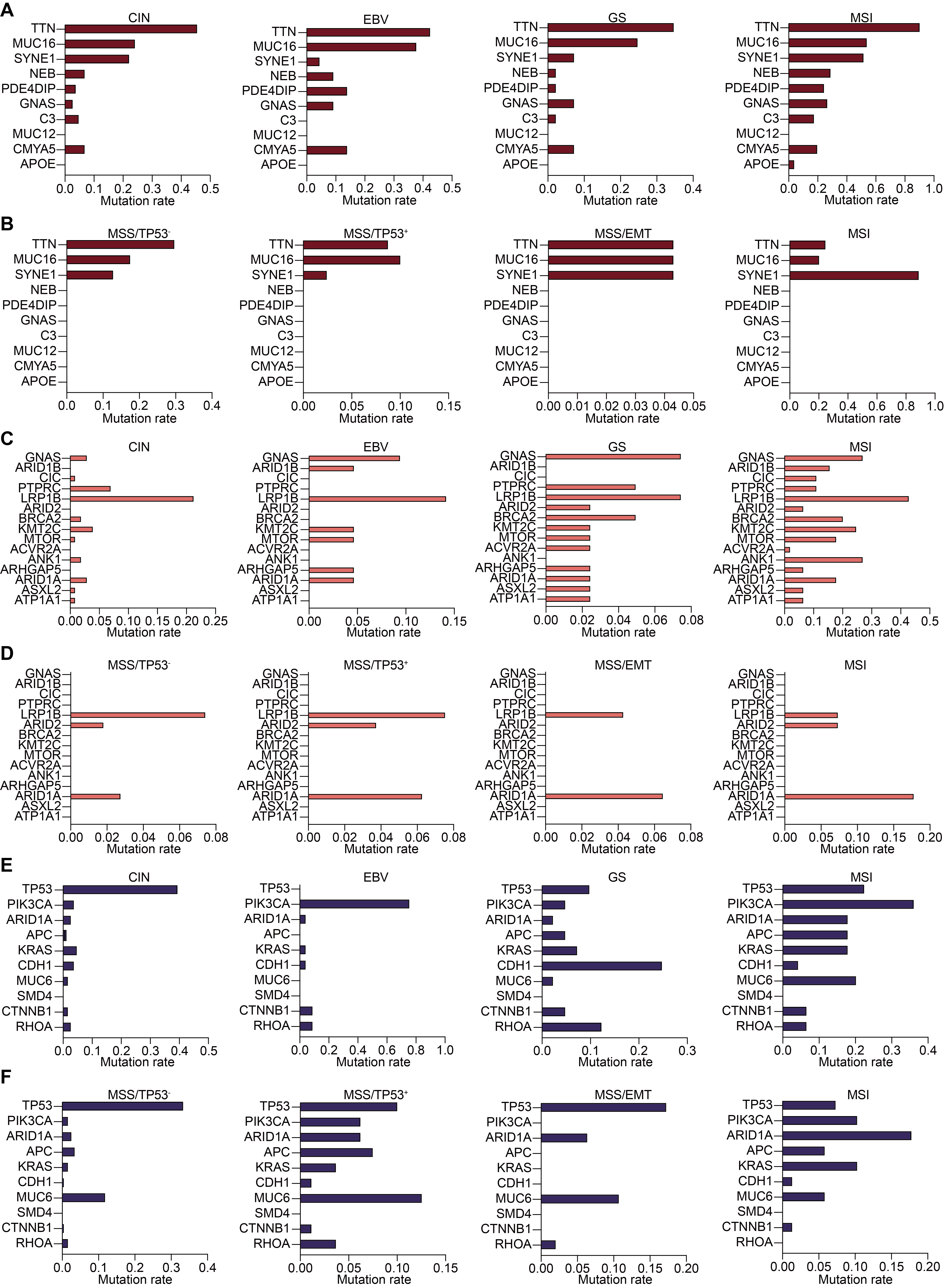

Supplement: Supplementary file 4 — Figure S4. (A) The non‐synonymous mutation rates of the top 10 SMGs in GA‐FG samples were shown in the four gastric cancers subtypes in the TCGA cohort. (B) The non‐synonymous mutation rates of the top 10 SMGs in GA‐FG samples were shown in the four gastric cancers subtypes in the ACRG cohort. (C) Mutational frequencies of the top 15 driver genes in GA‐FG samples were shown in the four gastric cancers subtypes in the TCGA cohort. (D) Mutational frequencies of the top 15 driver genes in GA‐FG samples were shown in the four gastric cancers subtypes in the ACRG cohort. (E) Mutational frequencies of the top 10 driver genes in TCGA cohort were shown in the four gastric cancers subtypes in the TCGA cohort. (F) Mutational frequencies of the top 10 driver genes in TCGA cohort were shown in the four gastric cancers subtypes in the ACRG cohort. [file CAM4-13-e70290-s003.png]

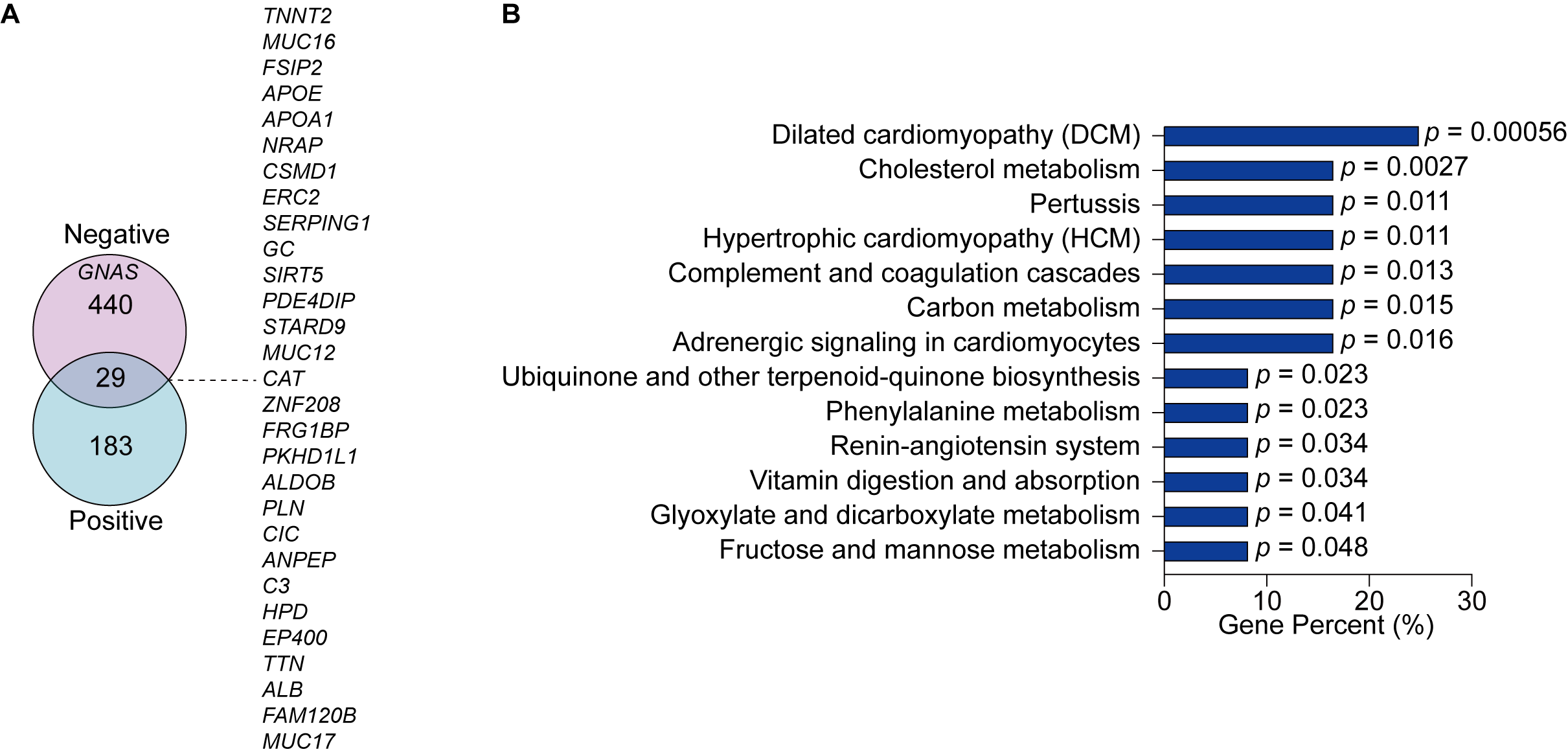

Supplement: Supplementary file 5 — Figure S5. (A) SMGs in GA‐FG patients with or without H. pylori infection were analyzed using the Maftool package. SMGs simultaneously profiled in GA‐FG patients regardless of H. pylori status were showed. (B) Signaling pathways in GA‐FG patients with or without H. pylori infection were presented based on the gene percentage. [file CAM4-13-e70290-s008.tif]
